# A wavelet-based VCG QRS loop boundaries and isoelectric coordinates detector

**DOI:** 10.3389/fphys.2022.941827

**Published:** 2022-10-21

**Authors:** Jan Kijonka, Petr Vavra, Pavel Zonca, Marek Penhaker

**Affiliations:** ^1^ Department of Cybernetics and Biomedical Engineering, Faculty of Electrical Engineering and Computer Science, VSB—Technical University of Ostrava, Ostrava—Poruba, Czechia; ^2^ Department of Surgical Studies, Faculty of Medicine of the University of Ostrava, Ostrava, Czechia; ^3^ Faculty of Electrical Engineering and Information Technology, University of Žilina, Žilina, Czechia

**Keywords:** vectorcardiography, QRS detection, segmentation, wavelet transform, isoelectric line detection

## Abstract

This paper deals with a wavelet-based algorithm for automatic detection of isoelectric coordinates of individual QRS loops of VCG record. Fiducial time instants of QRS peak, QRS onset, QRS end, and isoelectric PQ interval are evaluated on three VCG leads (X, Y, Z) together with global QRS boundaries of a record to spatiotemporal QRS loops alignment. The algorithm was developed and optimized on 161 VCG records of PTB diagnostic database of healthy control subjects (HC), patients with myocardial infarction (MI) and patients with bundle branch block (BBB) and validated on CSE multilead measurement database of 124 records of the same diagnostic groups. The QRS peak was evaluated correctly for all of 1,467 beats. QRS onset, QRS end were detected with standard deviation of 5,5 ms and 7,8 ms respectively from the referee annotation. The isoelectric 20 ms length PQ interval window was detected correctly between the P end and QRS onset for all the cases. The proposed algorithm complies the $\left({2\sigma }_{CSE}\right)$ limits for the QRS onset and QRS end detection and provides comparable or better results to other well-known algorithms. The algorithm evaluates well a wide QRS based on automated wavelet scale switching. The designed multi-lead approach QRS loop detector accomplishes diagnostic VCG processing, aligned QRS loops imaging and it is suitable for beat-to-beat variability assessment and further automatic VCG classification.

## Introduction

Analysis of the electrocardiogram (ECG) which is an image of the electrical cardiac activity is widely used method for diagnostics of many heart diseases. Automatic detection of fiducial points of cardiac cycle: QRS peak, P, QRS, T waves onsets and ends is essential for ECG diagnostics, signal processing and further automatic heart diseases classification. Isoelectric line detection allows ECG beats alignment in order to averaging and ectopic ECG beats evaluation (Gatzoulis et al., 2018) and ECG record intra-individual variability assessment (Matveev et al., 2007; Penhaker et al., 2014).

Besides the common 12-leads ECG imaging, the vectorcardiography (VCG) with its three (X, Y, Z) leads provides us three-dimensional imaging fully sufficient for description of cardioelectric space (Pavlov and Abel, 1975; Burch, 1985). Compared to common 12-leads ECG, the VCG is more advantageous method for computer processing due to fewer signals containing no redundant information and more accurate with corrected orthogonal leads, although clinical use is not common yet (Malmivuo and Plonsey, 1995). In both cases, the diagnostic information is evaluated from the characteristic P, QRS, T waves of common 12 ECG or 3 VCG leads.

We can find different approaches for automatic QRS detection and P, QRS, T waves segmentation based on the first and second derivative (e.g., Pan-Tompkins algorithm (Pan and Tompkins, 1985; Arzeno et al., 2006)), low-pass differentiation (LPD) (Chazal and Celler, 1996; Laguna et al., 1994), adaptive filtering (Soria-Olivas et al., 1998), wavelet transform (WT) (Martínez et al., 2004; Mahmoodabadi et al., 2005; Sahambi et al., 1997), morphology and gradient (TDMG) (Mazomenos, 2012), dynamic time warping (DTW) (Vullings et al., 1998a) or artificial neural networks (Dokur et al., 1997). An appropriate algorithm selection depends on a few factors like real-time (Pan and Tompkins, 1985; Mazomenos, 2012; Guven et al., 2014) or offline data processing (Martínez et al., 2004; Mahmoodabadi et al., 2005; Vullings et al., 1998a; Dokur et al., 1997), requirements for robustness of the detector in evaluation of records of purely healthy or also pathological cases, ability to recognize noisy signals with motion and other artifacts, processing of a standard quality or a diagnostic high resolution and high sampling rates ECG records, including single or multi-leads approaches.

Featured work follows on from the previous study of intra-individual variability of VCG record evaluation (Penhaker et al., 2014), where VCG loops were spatially aligned based on manually annotated isoelectric coordinates by cardiologist, time synchronized by QRS peaks and compared on specified QRS length (global QRS boundaries) for the intra-individual variability assessment. The proposed algorithm automates the process of the isoelectric coordinates and global QRS boundaries detection and follows the QRS peak detector based on wavelet transform and biorthogonal wavelet presented in previous work (Penhaker et al., 2014), as an alternative to Daubechies wavelet used in studies (Martínez et al., 2004; Mahmoodabadi et al., 2005; Sahambi et al., 1997). The properties of the biorthogonal wavelets were found ideally suited for ECG parameters estimation in (Sivannarayana and Reddy, 1999) since they excite various morphologies of ECG’s better at different scales. The wavelet scales for the proposed algorithm were experimentally established for QRS complex detection (QRS onset and QRS end) including wide-QRS conditions assessment detected on a different scale unlike previous studies (Martínez et al., 2004; Mahmoodabadi et al., 2005; Sahambi et al., 1997) using the same scales for various QRS morphologies. The QRS peak, QRS onset and QRS end detection is performed based on standardly used techniques of searching for maxima, minima, zero crossings and energy of wavelet coefficients in combination of time-domain signal analysis for QRS onset and QRS end adjusting by slope performed on a temporal search window. A time-domain signal analysis technique was already used in TDGM algorithm (Mazomenos, 2012) including signal preprocessing, QRS complex feature extraction employing Pan-Tompkins detection method (Pan and Tompkins, 1985) and temporal search windows followed by P-wave and T-wave feature extraction. While WT is quite robust in the presence of noise and baseline wander situations (Sivannarayana and Reddy, 1999; Dinh et al., 2001), the TDGM performed better in intricate ECG morphologies (Mazomenos, 2012). To benefit from both techniques, the proposed algorithm uses signal preprocessing before applying WT followed by time-domain-analysis. The signal preprocessing is performed by digital finite impulse response (FIR) high pass filter with 1 Hz cuttoff frequency in passband (0 dB signal attenuation) and infinite impulse response (IIR) 50 Hz or 60 Hz notch filter used for powerline distorted signals, meeting requirements for diagnostic ECG frequency bands (Klingfield et al., 2007; Medteq, 2022). The isoelectric line is searched on a temporal window localized before QRS onset as a flattest interval of a PQ segment, where the PQ segment appears to be the most acceptable location of zero cardiac activity, which is suitable for most pathological cases (Guven et al., 2014). In case of VCG leads, the isoelectric lines of the (X, Y, Z) leads defines coordinates with zero electrical activity situated in origin of the Cartesian coordinate system. With this assumption, the spatiotemporal VCG QRS loops alignment is performed by QRS loop isoelectric coordinates offsets removal and ECG beats synchronized with QRS peaks detected in one of the X , Y , or Z leads (Matveev et al., 2007). To compare individual QRS loops of a record, time-intervals to the left and to the right of the synchronization QRS peak are evaluated. In proposed algorithm, QRS global boundaries common for all QRS loops of a record are computed based on QRS onsets and QRS ends as number of samples to the left of the QRS peak ( boundL ) and to the right of the QRS peak ( boundR ).

The algorithm was developed and optimized on 161 records of 58 healthy control subjects (HC), 69 patients with myocardial infarction (MI), and 34 patients with bundle branch block (BBB) of the PTB (Physikalisch-Technische Bundesanstalt) diagnostic database of 12 standard ECG leads and 3 Frank VCG leads. All recordings are 2 min long, sampled at 1,000 Hz (Matveev et al., 2007; Bousseljot et al., 1995). The diagnostic ECG data are processed offline based on 3 VCG leads increasing robustness of the detector. Validation of the QRS detector and QRS onset and QRS end time instants detection was performed on CSE (Common Standards for Quantitative Electrocardiography) multilead database dataset 3 with 12 standard leads and 3 Frank leads of 125 biological ECGs. All recordings are 10 s long sampled at 500 Hz (Goldberger et al., 2000).

## Used methods

One of possible signal analysis is its comparison with a set of the test functions, where $Q=\left\{\varphi_{q}=e^{i\omega t},\omega \in R\right\}$ is set of test functions for well-known Fourier transform (FT), that contain all the dilatations and reductions of the periodic function $e^{it}$ by the factor ω (Willems et al., 1985). Another option for the time-frequency analysis is application of the WT similar to the FT. While the FT decomposes signal into a series of sine waves of different frequencies, WT decomposes signal into the “wavelets”, dilated and translated versions of the so-called Mother wavelet ψ(t) . Wavelet is nonzero only at the finite time interval, or the values outside the interval are negligibly small. Consequently, whatever value of the spectrum is used, based on the wavelet, it is influenced only by the corresponding time interval of the analysed signal. Wavelet basis functions cover the entire time span of the analysed signal in parts. Therefore, the full information is preserved. Compared to the smooth sinusoidal curve of an infinite length, wavelet is compact and irregular shaped. With these features, wavelet is an ideal tool for unsteady signals with discontinuities and sharp changes analyses and possibilities of localization in time (Aldroubi and Unser, 1996).

### Continuous wavelet transform

If the base fiction Q={ψ((t−b)/a),(a,b)∈(0,∞)×R} is used, we obtain the continuous wavelet transform $\left(W_{\psi }f\right)\left(a,b\right)$ of the signal $f\left(t\right)\in L_{2}\left(R\right)$ (Aldroubi and Unser, 1996):

$$\left(W_{\psi }f\right)\left(a,b\right)=\int_{-\infty }^{\infty }{\left|a\right|}^{-1/2}f\left(t\right)\bar{\psi \left(\frac{t-b}{a}\right)}dt$$ (1)

where a is so-called dilatation scaling parameter, b is translation parameter, $\bar{\psi }$ is complex conjugate of ψ and where $\psi \in L_{2}\left(R\right)$ is mother wavelet—oscillation function.

Continuous wavelet transform (CWT) uses a sampled data, but the process of translation is a smooth operation across the length of the sampled data. The scaling can be defined from a minimum (original signal scale) to a maximum selected by the user, making a finer resolution possible. The disadvantage compared to discrete wavelet transform (DWT) is increasing of the computational time and higher memory requirement for wavelet coefficients calculation (Aldroubi and Unser, 1996).

### Wavelet type used

**FIGURE 1:**
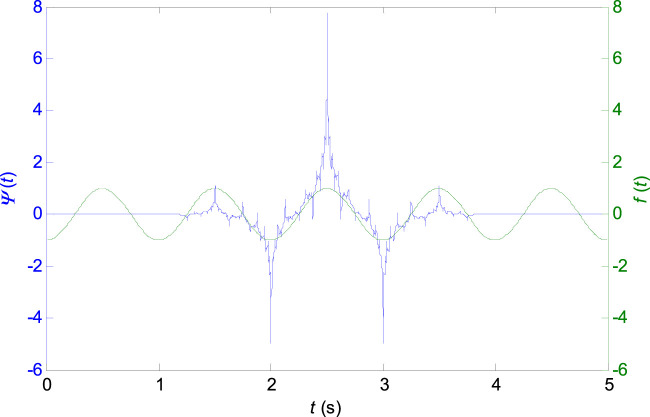
Biorthogonal wavelet 2.2 with the central frequency $f_{central}=1,0008\;Hz$ used in the presented QRS detector algorithm.

Wavelets can be classified as orthogonal, non-orthogonal and biorthogonal. Only biorthogonal wavelets provide the time symmetry and prevent phase shifts of the transformed signal (Altmann, 1996). For the ECG signal analysis, the shape of the signal in the time-domain is important, while a signal reconstruction (inverse-transform) is not required. From this standpoint, the biorthogonal wavelets are advantageous for the ECG signal analysis. The shape of the biorthogonal wavelet 2.2 used in this work resembles the shape of the ECG waveform (see Figure 1) which excite various morphologies of ECG waveforms better at different scales (Sivannarayana and Reddy, 1999). Application of biorthogonal wavelets for the ECG parameters (P, QRS, and T) estimation is known from the literature (Sivannarayana and Reddy, 1999; Louis et al., 1997) and it was already used for QRS peak detection in the previous work (Penhaker et al., 2014). The central frequency of the biorthogonal wavelet 2.2: $f_{central}=1,0008\;Hz$ has a relation to scale a from the Eq. 1 and pseudofrequency at the scale a according to the relation (3) and Table 1, further explained in the next chapter 2.3.

**TABLE 1:** Pseudofrequencies for the scales a={10, 30, 50, 70, 120} , central frequency $f_{central}=1,0008\;Hz$ and sampling period ∆=0,001 s .

a(–)	$f_{a}\left(Hz\right)$
10	100
30	33.4
50	20
70	14.3
120	8.3

### Scalogram and coefficients of the continuous wavelet transform

**FIGURE 2:**
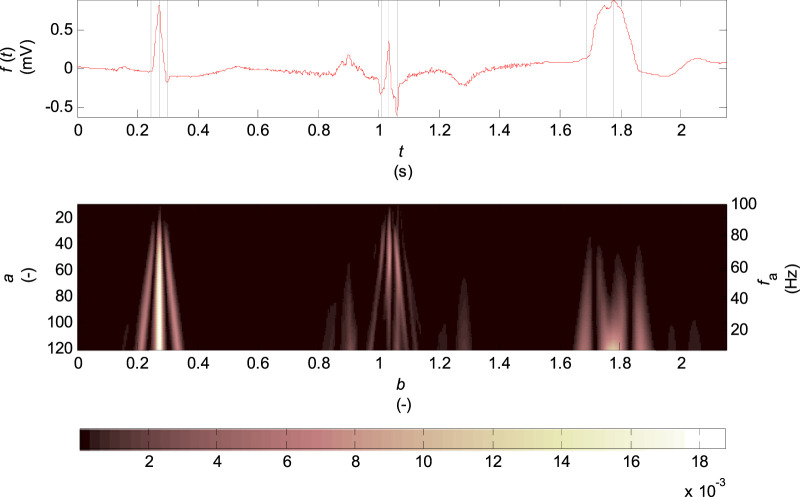
Scalogram of the continuous wavelet transform for biorthogonal wavelet 2.2, scale parameter a={10,…,120} and corresponding pseudofrequencies $f_{a}≅\left\{100,\ldots ,8\right\}\;Hz$ . The input signal is one lead VCG of one cardiac cycle of the named records of the diagnostic PTB database: from the left the “s0130lre” of a HC patient, the “s0235lre” of a MI patient, “the s0448_re” of a LBBB patient. The single Q, R, S waves are marked by dashed lines in the input signal.

In Figure 2 there is the scalogram of continuous wavelet transform for biorthogonal wavelet 2.2 which is used in this work. The input signal is one VCG lead of healthy control patient (HC), a patient with inferior myocardial infarction (MI) and a patient with the left bundle branch block (LBBB).

The scalogram represents the percentage of energy of wavelet coefficients at the given scale a and location b of the wave $\psi_{a,b}$ by the relation:

$$E_{p}\left(a,b\right)=\frac{{\left|\left(W_{\psi }f\right)\left(a,b\right)\right|}^{2}}{\overset{M}{\underset{i=1}{\sum }}\overset{N}{\underset{j=1}{\sum }}{\left|\left(W_{\psi }f\right)\left(a_{i},b_{j}\right)\right|}^{2}}∙100$$ (2)

where M is number of the scales, N is number of the translations corresponding to the number of samples of the input signal.

The scaling parameter is selected in the range of a={10,…,120} . For the related scales the corresponding pseudofrequencies are calculated (Kumar et al., 2018):

$$f_{a}=\frac{f_{central}}{a∙∆}$$ (3)

where $f_{a}\;\left(Hz\right)$ is pseudofrequency at the scale a , $f_{central}\;\left(Hz\right)$ is the central frequency of the wavelet, a is scale and ∆(s) is sampling period.

Among the scale and frequency there is only an approximate relationship since the wavelet contains other frequency components in addition to the calculated central frequency $f_{central}$ . The selected range of the scales was specified on the basis of the power spectrum of the QRS. To a frequency about 10 Hz the P and T waves spectra and the motion artefacts can be shown. The representation of the frequencies above 40 Hz is relatively low and the high-frequency interference is shown in this band. For the central frequency $f_{central}=1,0008\;Hz$ of the biorthogonal wavelet used (Figure 1) and the sampling period ∆=0,001 s there are computed the pseudofrequencies at the desired scales a={10, 30, 50, 70, 120} shown in the Table 1.

The scalogram (see Figure 2) shows the highest percentage of energy of coefficients concentrated at the R wave and Q, S wave times for the record of healthy patient in the entire range of scales a={10,…,120} . The record of patient with inferior MI has a small R wave amplitude considering the P, T waves and has a high-frequency noise. For this record, there appear brighter areas in time intervals of the T and P waves for the scale a>60 in the scalogram. The last record of patient with LBBB with a wide QRS complex and therefore with the higher proportion of lower frequencies has a relatively small percentage of energy for the scale <40 .

**FIGURE 3:**
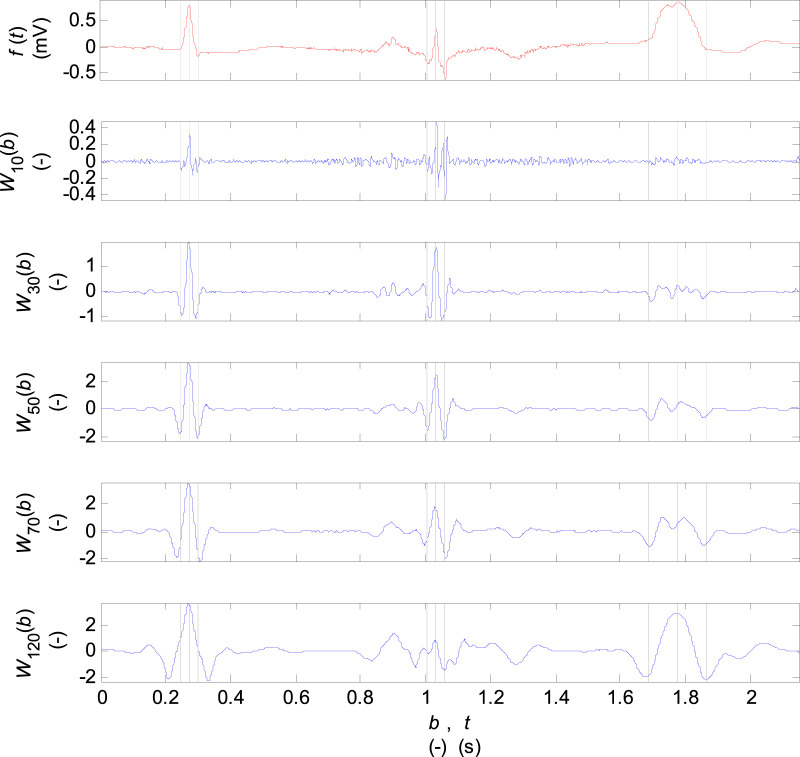
Coefficients of the wavelet transform for the scale parameter a={10, 30, 50, 70, 120} and the mother biorthogonal 2.2 wavelet. The input signal is one VCG lead of one cardiac cycle of the named records of the diagnostic PTB database: from the left the “s0130lre” of a HC patient, the “s0235lre” of a MI patient, “the s0448_re” of a LBBB patient. The single Q, R, S waves are marked by dashed lines in the input and transformed signals.

The coefficients of wavelet transform for discrete scale values a={10, 30, 50, 70, 120} are plotted in Figure 3. At the scale a=10 , there are relatively large frequency noise and low values of coefficients. At the scale a=30 and a=50 there are good localisations of Q, R, S waves with a small amplitude for the P and T waves. The higher scales are suitable for Q, R, S localisation for the records with a wide QRS.

## Algorithm description

**FIGURE 4:**
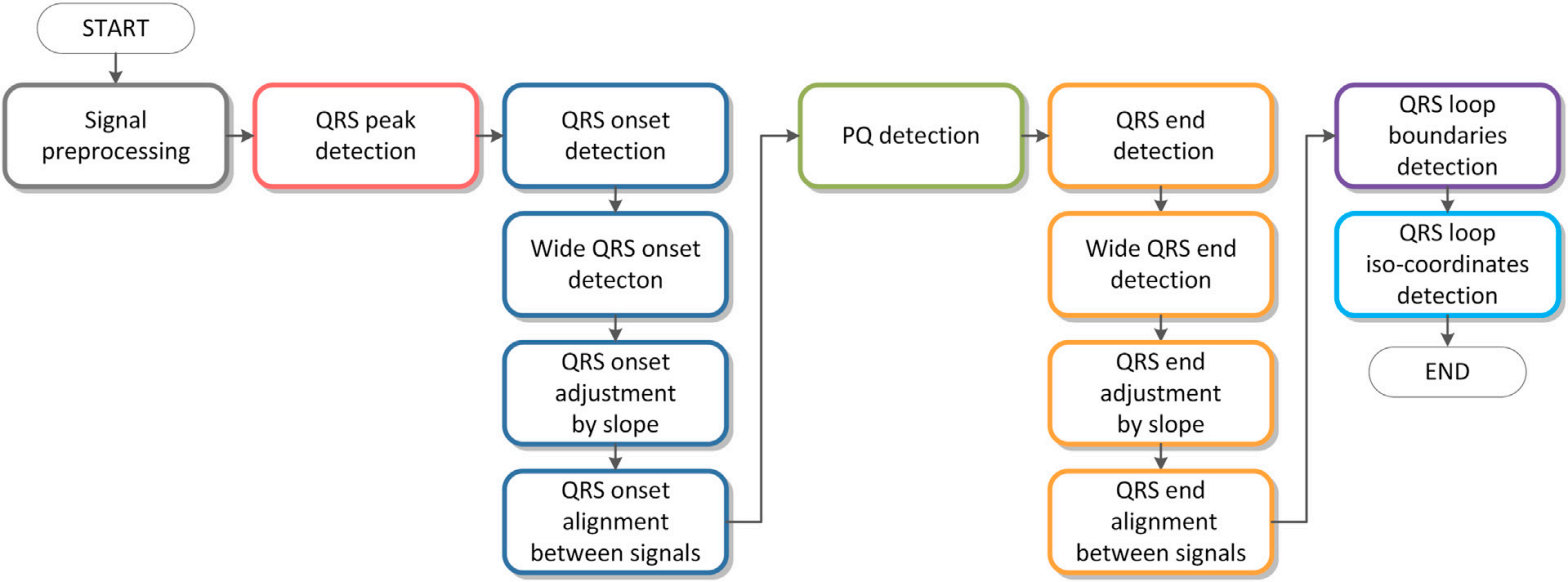
Algorithm flowchart.

**FIGURE 5:**
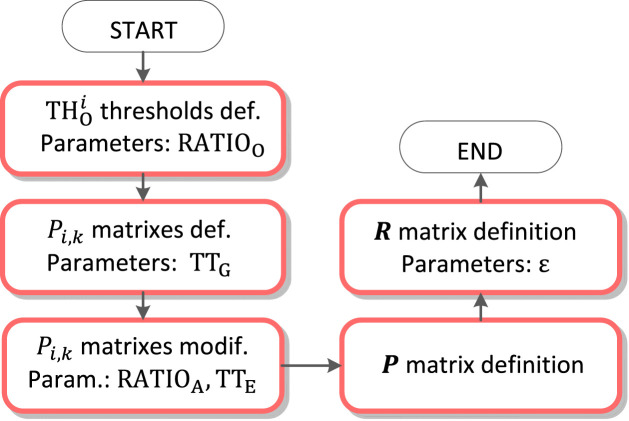
QRS detection flowchart.

**FIGURE 6:**
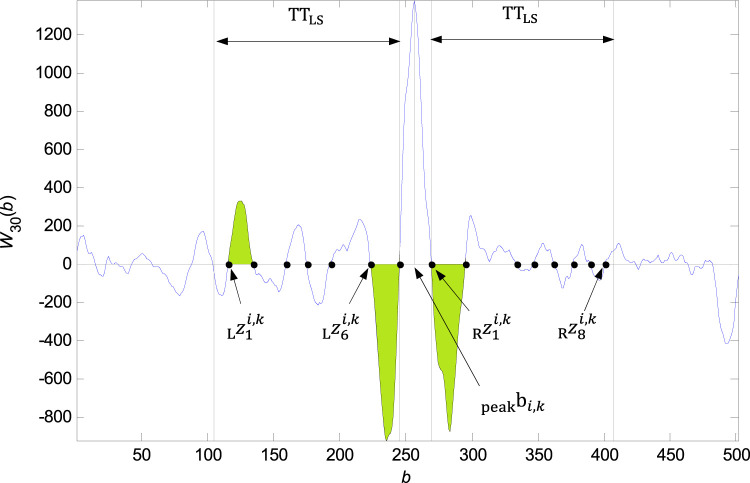
Founded zero points zsi,kL to the left of the bpeaki,k and zti,kR to the right of the bpeaki,k in the ${TT}_{LS}$ bounds. The zero points met the condition corresponding to the green denoted intervals and the elements {logicLsi,k}1α−1={1,0,0,0,0,1} and {logicRti,k}1β−1={1,0,0,0,0,0,0} .

**FIGURE 7:**
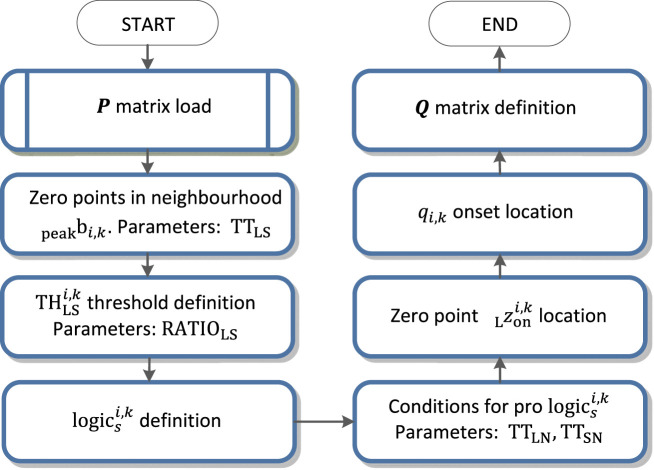
QRS onset detection flowchart.

**FIGURE 8:**
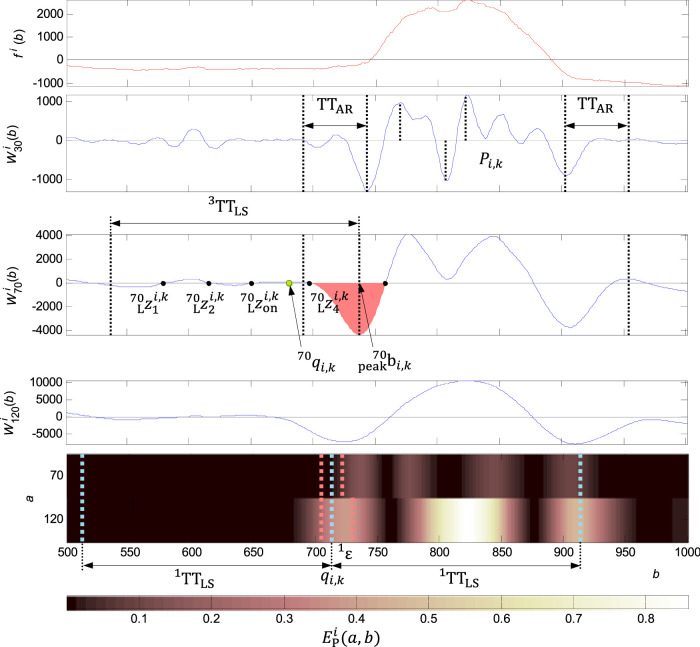
Adjusting the QRS onset for a wide QRS. In the first graph from the top: the original signal $f^{i}\left(b\right)$ (wide QRS). In the second graph from the top: $W_{30}^{i}\left(b\right)$ with the set of $P_{i,k}$ points found and delimited by the ${TT}_{AR}$ . In the third graph from the top: $W_{70}^{i}\left(b\right)$ and zero points from zL701i,k to zL704i,k detected in the range of TT3LS , the time interval of the zero point zL704i,k is coloured by red, where logic704i,k=1 , the new zero point zL70oni,k=zL703i,k found and the QRS onset q70i,k . In the fourth graph from the top: $W_{120}^{i}\left(b\right)$ for comparison to scalogram. In the fifth graph from the top: $E_{P}^{i}\left(70,b\right)$ and $E_{P}^{i}\left(120,b\right)$ and maximum energy Ei,kMAX≅0,81 in the range of TT1LS , the $q_{i,k}$ neighbourhood coloured by red is the zone for the high energy detection by the defined condition. In the ε1 neighbourhood there were not any thresholds for $E^{i,k}\left(a,b\right)$ exceeded, however, at the scale 120 there was the double energy related to the scale 70 in the range defined by TT1LS . On condition for the wide QRS was met and the QRS was qualified as wide.

**FIGURE 9:**
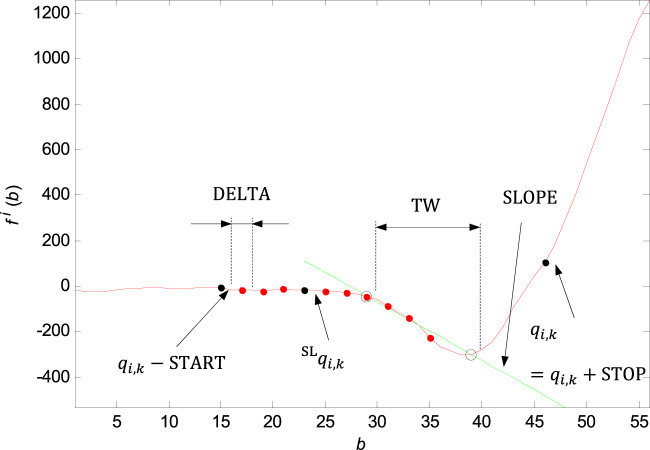
Marking of the shifting window with the width of TW , initial point in the $q_{i,k}-START$ point, end point in the $q_{i,k}=q_{i,k}+STOP$ point and shift step DELTA . In each window, there is the line slope SLOPE evaluated. In the case of the QRS onset and offset adjustment, the line slope is evaluated only from the initial and end points of the window. The new point qSLi,k marked in the graph is defined based on the ${TH}_{SLOPE}$ parameter.

**FIGURE 10:**
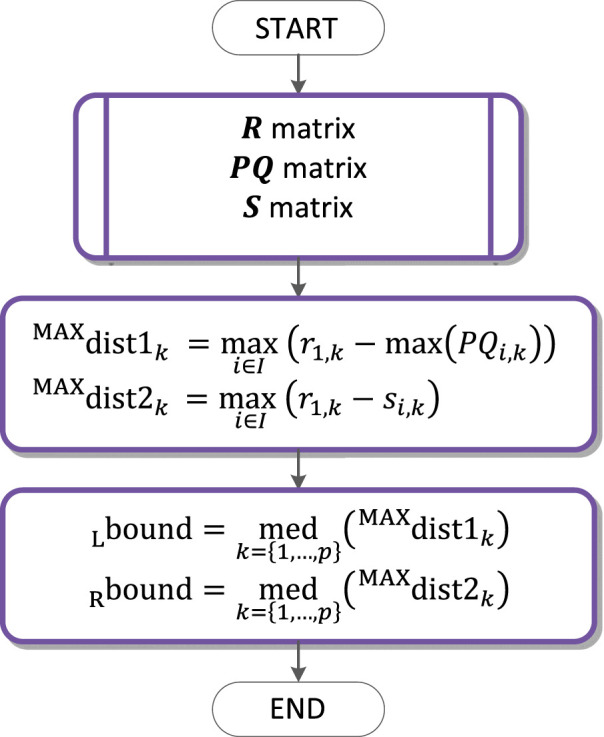
QRS loop boundaries detection flow chart.

**FIGURE 11:**
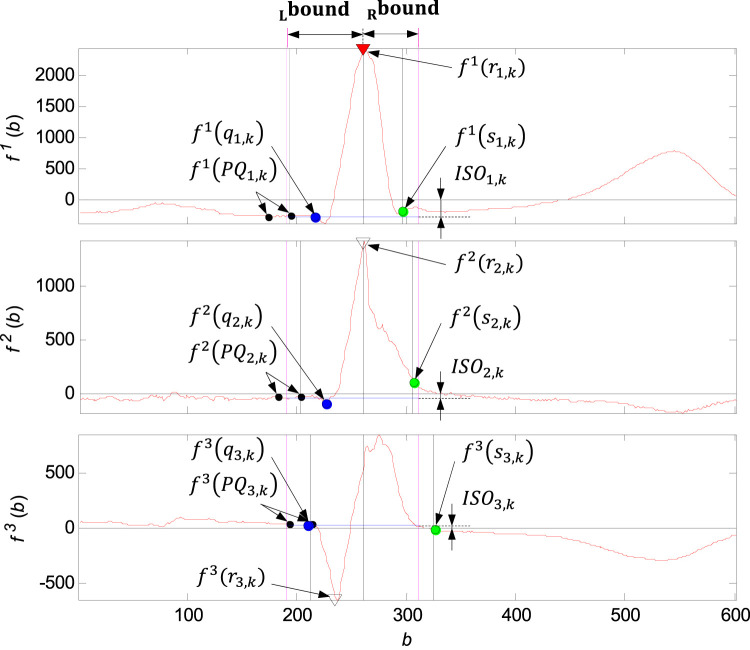
Illustration of the QRS loop boundaries detection and the individual time instants $q_{i,k}$ , $r_{i,k}$ , $s_{i,k}$ , ${PQ}_{i,k}$ . The function value in the synchronising point $r_{1,k}$ is marked by the red triangle, next there are marked the function values in the points $r_{2,k}$ , $r_{3,k}$ (empty triangle), function values in the points $q_{i,k}$ of the QRS onsets (blue circle), function values in the points $s_{i,k}$ of the QRS ends (green circle), function values in the points of the ${PQ}_{i,k}$ sets of the PQ segments (black circle). The boundaries of the QRS loop boundL , boundR marked with violet colour. Isoelectric coordinates ${ISO}_{i,k}$ marked with blue isolelectric lines.

A flowchart of the algorithm is shown in Figure 4. Individual parts of the algorithm are described in figures from Figures 5–10. The input signals of the algorithm are VCG leads ( X, Y, Z ) with indexes i∈I={1,2,3} , samples of the signal record are marked as b∈B={1,…,N} , where N is the total number of samples. Functional values of the input signals are marked as $f^{i}\left(b\right)$ in units GAIN∙mV , where GAIN=2000 is gain of the signal. The signals are sampled with the sampling frequency $f_{s}=1000\;Hz$ . The outputs of algorithm are the time instants of the QRS peak, QRS onset and QRS end, and PQ interval onset and offset for each QRS complex and each signal of the VCG record. These time instants are stored in the matrixes R , Q , S , PQ , where the indexes i,k of each matrix element corresponds to the signal index and the sequential number of the heartbeat detected. Another output is the QRS loop global boundaries marked as boundL and boundR . The boundaries indicate the number of samples on left and right to the synchronizing QRS peaks detected, evaluated globally for a record. As the synchronizing wave the QRS peak time instant in the lead X is selected. An example of QRS boundaries and isoelectric lines detection is in Figure 11.

### Signal preprocessing

The VCG leads ( X, Y, Z ) signal preprocessing is performed to remove noise added from powerline interference and baseline wander caused by respiratory and motion artefacts. The baseline wander removal is accomplished by digital FIR high pass filter with 1 Hz cuttoff frequency in passband (−0 dB) and 0,5 Hz cutoff frequency in stopband (−61 dB). The properties of the FIR filter type used allows to preserve useful ECG frequency bands with no additional signal distortion, meeting requirements of American Heart Association (AHA) (Klingfield et al., 2007) for diagnostic ECG. The powerline interference is filtered by notch 50 Hz or 60 Hz filter depending on the presence of the interfering signals. Used 0.17 Hz narrowband notch with an attenuation greater than 60 dB affects the original signal only in the narrow frequency band around the 50 Hz or 60 Hz interference with a negligible effect on the useful ECG frequency band (Klingfield et al., 2007), (Medteq, 2022).

### QRS peak detection

QRS detection is based on waletet transform of input signals on scale a=30 (see Figure 3). At first, the threshold ${TH}_{O}^{i}$ of wavelet transform function $W_{30}^{i}\left(b\right)$ for each signal i∈I is defined, where parameter ${RATIO}_{O}=0,7$ is the oscillation ratio of the absolute maximum value of W30iκ(b) function. Then, the sets $P_{i,k}$ are formed, where each set represents the estimated time instants of the heartbeat. The criterion for the $P_{i,k}$ sets definition is the parameter ${TT}_{G}=200\;samples$ , which indicates the maximum number of samples between the two subsequent heartbeats. From each set of the bmaxi,k∈Pi,k points with the function values lower then ${RATIO}_{A}=0,7$ are excluded. Then the comparison of the QRS detected (represented by $P_{i,k}$ sets) between signals is done. If the QRS is detected in a single lead then the QRS (corresponding $P_{i,k}=\emptyset$ ) is excluded. If the QRS is detected in two of three leads, the new point of QRS detection is added to the corresponding $P_{i,k}$ set. All the QRS detected at the edge of the record within the limits ${TT}_{E}=200\;samples$ of the record are excluded. Then the matrix P with the elements bpeaki,k is created, where bpeaki,k is one element from each $P_{i,k}$ set defined as the maximum function value of the |W30i(bmaxi,k)| . In the neighbourhood of the point bpeaki,k , where the neighbourhood of the point is defined as ε=10 samples , the maximum amplitude of the function $\left|f^{i}\left(b\right)\right|$ is found. This point indicates the time instant of the QRS occurrence and it is added to the R matrix. Simplified flowchart of the QRS peak detection is in Figure 5.

### QRS onset and QRS end detection

Detection of the QRS onset and QRS end is based on the zero crossing of the function $W_{30}^{i}\left(b\right)$ . ∀(i,k) zero points in the neighbourhood of bpeaki,k are searched for, where bpeaki,k are elements of the P matrix. The bpeaki,k neighbourhood is defined by the parameter ${TT}_{LS}=150\;samples$ . In the left neighbourhood of the bpeaki,k the zero points zsi,kL are searched for, where s∈{1,…,α} , α is the number of zero points in the left neighbourhood of the bpeaki,k . In the right neighbourhood of the bpeaki,k zero points zti,kR are searched for, where t∈{1,…,β} , β is the number of zero points in the right neighbourhood of the bpeaki,k . ∀(i,k) , there is defined the oscillation threshold ${TH}_{LS}^{i,k}$ of the function $W_{30}^{i}\left(b\right)$ by the parameter ${RATIO}_{LS}=0,3$ .

For the QRS onset detection, there is the partial function ${logic}_{s}^{i,k}$ defined, where for each zero point zsi,kL , where s∈{1,…,α−1} is the function value 1 assigned if the condition W30i(zsi,kL≤b≤zs+1i,kL)>THLSi,k is met or the function value 0 is assigned if the condition is not met.

An example of zero points in the bpeaki,k neighbourhood finding and the ${logic}_{s}^{i,k}$ definition is shown in the Figure 6. The elements of ${\left\{{logic}_{s}^{i,k}\right\}}_{1}^{\alpha -1}$ is the sequence of values 1 and 0 , where 1 corresponds to the suitable interval of the signal $W_{30}^{i}\left(b\right)$ and 0 correspond to the non suitable interval, where zsi,kL≤b≤zs+1i,kL , s∈{1,…,α−1} . There are the rules for the QRS onset zoni,kL detection established:

• If the length of one last non suitable interval is lower then ${TT}_{SN}=10\;samples$ , the interval is assigned as the suitable one.
• As the zero point zoni,kL of the QRS onset, the zero-point located after couple of the unsuitable intervals is assigned or the zero-point located after an unsuitable interval with the length higher then ${TT}_{\mathrm{LN}}=25\;samples$ is assigned.

The QRS onset is than accurately traced in the interval between the zero points zoni,kL and zon+1i,kL . The criterion for the QRS onset assignment is the signal shape of the function $W_{30}^{i}\left(b\right)$ in the right neighbourhood of the zoni,kL . The founded QRS onset is put into the matrix Q . The flowchart of the QRS onset detection is in Figure 7.

The QRS end is detected by analogy in the right neighbourhood of the bpeaki,k . The founded QRS end is put in the matrix S .

### Wide QRS onset and QRS end detection

Adjusting the QRS onset or QRS end is performed in the case of meet the condition of exceeding the percentage of energy of the wavelet coefficients 2) in the QRS onset or QRS end neighbourhood. It is compared the energy percentage at scales a=70 and a=120 in the time window width, given by the parameter TT1LS=200 samples and the percentage of energy at these scales in the ε=16 samples1 neighbourhood for the QRS onset, and TT2LS=200 samples and ε=24 samples2 neighbourhood for the QRS end. The adjusting of the QRS onset and QRS end is based on the zero crossing of the function $W_{70}^{i}\left(b\right)$ , when the similar process as for the QRS onset and QRS end detection is kept. ∀(i,k) there are the zero points zsi,kL70 and zti,kR70 found in the neighbourhood bpeak70i,k , given by the parameter TT3LS=200 samples . The bpeak70i,k are the points with the local maximum of the function $\left|W_{70}^{i}\left(b\right)\right|$ where $b\in \left\{\min\left(P_{i,k}\right)-{TT}_{AR}\leq b\leq \max\left(P_{i,k}\right)+{TT}_{AR}\right\}$ . Parameter ${TT}_{AR}=50\;samples$ . An example for the QRS onset adjustment for the wide QRS is shown in Figure 8.

### QRS onset and QRS end adjustment by slope

QRS onset and QRS end adjustment by slope is based on the linear regression method and line slope calculation on the time window of the input signal in the section before the QRS onset ( $q_{i,k}$ point), and after the QRS end ( $s_{i,k}$ point).

For the QRS onset adjusting there are some parameters set: the window width TW=10 samples , the initial point of the investigation—number of samples before the $q_{i,k}$ , START=30 samples , the end point of the investigation—number of samples after the $q_{i,k}$ , STOP=0 samples , the movement step of the window, DELTA=2 samples . The line slope ${SLOPE}_{s}^{i,k}$ is evaluated based on the linear regression of the initial and end points of the time window. The threshold for the slope exceeding is set to ${TH}_{SLOPE}=10$ . If the slope ${SLOPE}_{s}^{i,k}$ is exceeded for s>3 , where s is the accumulate shift of the window s∈{0,DELTA, 2⋅DELTA,…, START+STOP−TW} , then the first window in the sequence with the slope ${SLOPE}_{s}^{i,k}<{TH}_{SLOPE}$ is found and the new QRS onset is defined. If there is not any time window that met the condition, the new QRS onset is defined in the point qSLi,k=qi,k−START . The QRS onset adjustment by slope is shown in Figure 9.

Finding the slope in the right neighbourhood of the $s_{i,k}$ and finding the new QRS end sSLi,k is carried out analogously.

### QRS onset and QRS end alignment between signals

Aligning of the QRS onsets or QRS ends between the three VCG leads is performed in case of exceeding the distances between the $q_{i,k}$ , or $s_{i,k}$ points for the given k and triple of signals i∈I by the defined threshold ${TT}_{BS}=80\;samples$ . If the condition is met, the farthest point is shifted on the mean value of the remaining two points and the new QRS onset qALi,k or QRS end sALi,k is found.

### PQ detection

PQ isoelectric interval detection is based on linear regression and line slope evaluation on the temporal search window of the input signal in the neighbourhood of the QRS onset point $q_{i,k}$ . By the experimental work of the PQ detector, there were any other parameters evaluated on the time window (line slope difference, the mean value of the signal, standard deviation difference). However, only one parameter—the line slope was chosen for the algorithm. The time window is given by parameters: the window width TW1=20 samples , the initial point of the investigation—the number of samples before the $q_{i,k}$ , START1=50 samples , the end point of the investigation—number of samples after the $q_{i,k}$ , STOP1=10 samples , the movement step of the window, DELTA1=2 samples . The line slope ${SLOPE}_{s}^{i,k}$ is evaluated based on the linear regression of the function $f^{i}\left(b\right)$ on the time window. The time window with the minimum line slope is selected and defined as looked-for part of the PQ segment. Although the PQ segment is usually longer than TW1 , its entire interval is known as isoelectric. For this reason, it is sufficient to choose only the suitable part of the PQ segment for isoelectric line evaluation. The beginning and end of the time window founded are put into the ${PQ}_{i,k}$ set and into the PQ matrix**.**

### QRS loop boundaries detection

The QRS loop boundaries detection is performed based on the QRS peak detected ( R matrix), the PQ segment detected ( PQ matrix) and the QRS end detected ( S matrix). ∀(k) the maximum distance of the ${PQ}_{i,k}$ and $r_{1,k}$ is computed, where $r_{1,k}$ is the time instant of the QRS detected in the lead X , specified as a synchronising wave for all the three leads X, Y, Z and given k . Median of these distances is assigned as boundL —the left boundary of the QRS, its value is the number of samples to the left of the synchronising wave. ∀(k) there is computed the maximum distance of $s_{i,k}$ and $r_{1,k}$ . Median of these distances is assigned as boundR —the right boundary of the QRS loop, its value is the number of samples to the right of the synchronising wave, where boundL and boundR are identical for all k (all the QRS loops of the record). The flowchart of the QRS loop boundaries detection is in Figure 10.

### QRS loop isoelectric coordinates detection

Isoelectric coordinates are evaluated for each of the QRS loop k∈{1,…,p} of the record based on the mean voltage levels in the PQ intervals detected for the signals i∈I from the relation (4):

$${ISO}_{i,k}=\underset{\min(PQ_{i,k})<b<\;\max(PQ_{i,k})}{mean}\left(f^{i}\left(b\right)\right)$$ (4)

where k is the sequence number of QRS loop detected, i is index of the signal, $f^{i}\left(b\right)$ is the input signal, b is sample of the signal, ${PQ}_{i,k}$ is set of beginning and end point of the PQ interval, mean() is a mean value.

## Results

Validation of the QRS detector and QRS onset and QRS end time instants was performed on CSE multilead database dataset 3 with 12 standard leads and 3 Frank leads of 125 biological ECGs. All recordings are 10 s long sampled at 500 Hz with 1 µV resolution. Patients with various diagnoses including bundle branch blocks and aspecific conduction defects with significant changes in ECG image causing a wide QRS (>120 ms) was observed in 21 of total of 125 cases. Number of cases for four main groups of diagnoses can be seen in Table 2 (Goldberger et al., 2000), (Zhao et al., 2004), (Willems et al., 1990).

**TABLE 2:** Preview of CSE validation database diagnoses.

Diagnose	Number of cases
Healthy control	33
Bundle branch blocks and fascicular blocks	33
Myocardial infarction	33
Other	26
**Total**	125

To use the records sampled a at 500 Hz, the records were firstly resampled to 1,000 Hz in the context of preserving original filters and frequency scales of wavelet transform. Form the total of 125 records, one record with an artificial pacemaker was excluded.

*Median referee* annotation based on five referee cardiologists and *median program* annotation based on nine different ECG analysis programs are provided in CSE database together with ECG waveforms in digital data file format. The cardiologists only analysed every fifth record and additionally some waves, for which a set of analysis programs differed significantly. For this study, the records were divided into two groups, where in the first group the results were compared with median referee annotation, while for the second group only the median program annotation was available. However, the median wave recognition results of the nine ECG analysis programs are almost identical to the final visual estimates obtained by the referees and thus can be used as a substitute of the reference annotation (Zhao et al., 2004).

In the CSE database we obtained 3 different sets of annotations, one for each channel (X,Y,Z) . For selecting a single location for each characteristic point, we used a rule consisting of ordering the 3 single-lead annotations and selecting as the onset (end) of QRS the first (last) annotation whose k=1 nearest neighbor lay within smallest δ ms interval. This rule had been already used in (Chazal and Celler, 1996), (Laguna et al., 1994), (Martínez et al., 2004), where values k=2 to k=3 and δ=6 ms to δ=10 ms were used for 12 or 15 single leads annotations.

To evaluate the QRS peak detector, we use the sensitivity ( Se ) and positive predictivity ( $P^{+}$ ) formulas:

$$Se=\frac{TP}{TP+FN};\;P^{+}=\frac{TP}{TP+FP}$$ (5)

where TP is number of true postive detections, FN is number of false negative detections and FP is number of false positive misdetections

For total of 1,467 beats, we obtained a sensitivity of Se=100% and a positive predictivity of $P^{+}=100\%$ .

To assess QRS onset and QRS end time delineation performance, mean value μ and standard deviation σ of time differences between presented algorithm and reference annotation were calculated. The algorithm accomplishes the two standard deviations $\left({2\sigma }_{CSE}\right)$ tolerances (“loose criteria”) for both QRS onset and QRS end time evaluation (see Table 3), where the $\left({2\sigma }_{CSE}\right)$ criteria is a robust estimation of the “median reader” of what can be expected from an expert cardiologist (Willems et al., 1990). This criterion was also used in (Laguna et al., 1994; Martínez et al., 2004; CSE WORKING PARTY, 1985; Vila et al., 2000), while for others (Chazal and Celler, 1996), the one standard deviation criteria (“strict criteria”) should be attained. The delineation results comparison between LPD methods presented in (Chazal and Celler, 1996; Laguna et al., 1994) and WT methods presented in (Martínez et al., 2004; Sahambi et al., 1997) in the CSE database are already available in (Martínez et al., 2004), where the proposed algorithm achieves comparable or better results for the QRS onset and QRS end detection than others except for (Sahambi et al., 1997), where lower error standard deviation is reported. However, we have no information about what dataset and what one-lead to multilead rule were used.

**TABLE 3:** ORS onset and QRS end delineation performance.

References	QRS onset (#) μ±σ (ms)	QRS end (#) μ±σ (ms)
*Median referee* annotation	(32) 3.0 ± 5.6	(27) -0.1 ± 7.9
*Median program* annotations	(92) 0.9 ± 5.4	(97) 1.7 ± 7.7
**Combined**	**(124) 1.4 ± 5.5**	**(124) 1.3 ± 7.8**
**Tolerances** $\left({2\sigma }_{CSE}\right)$ (Willems et al., 1990)	**6.5**	**11.6**

To evaluate the isoelectric PQ segments detection accuracy, a condition was established: The PQ interval of the temporal 20 ms window must be located between the end of the P wave and the beginning of the QRS onset annotated by a referee. The condition was met for all the 124 records.

Requirements for the global boundL and boundR QRS boundaries detection, calculated by relations (see Figure 11) were to include all QRS loops of the record between these two bounds with respect to intra-individual variability of the record (Penhaker et al., 2014). The boundL detected in isoelectric PQ segment and boundR detected in the most probably QRS end was ideal for QRS loops comparing and visualisation.

## Conclusion

Designed QRS loop detector evaluates zero crosses of the wavelet transform, linear regression and percentage of energy of the wavelet coefficients on specified scales and uses the experimentally set logical terms to select an appropriate interval between zero crossings to QRS onset and QRS end evaluation. To provide a finer resolution an accurate localisation in time, a continuous WT and biorthogonal wavelet *versus* quadratic spline in other studies is used. To adjust fiducial points, the problem is transferred to time-domain on pre-defined temporal windows. The PQ isoelectric interval is found on temporal search window as the flattest part of the PQ segment.

Detected isoelectric coordinates of the individual QRS loops of a VCG record allows the spatiotemporal QRS loops alignment synchronized by QRS peaks with the length of QRS determined by automatically detected QRS bounds of the record. The spatiotemporal QRS loops alignment allows the QRS loops comparison, averaging, ectopic QRS loops evaluation and intra-individual variability assessment which was addressed in the previous study.

Compared to other algorithms, the proposed solution combines both WT as a robust method in the presence of noise as well as in baseline wander situations, and time-domain techniques for better performance in intricate ECG morphologies to adjust QRS onset, QRS end, and isoelectric PQ interval detected on temporal search window. Special care is given to wide pathological QRS, where fiducial points are evaluated on different scale for experimentally set conditions. The algorithm uses multilead approach, where three VCG leads are used simultaneously for detection of QRS onset and QRS end.

The results of QRS peak, QRS onset and QRS end detection was compared with other published approaches and have shown that the developed algorithm provides a reliable and accurate delineation of the ECG signal better or comparable with other algorithms with standard deviation complying $\left({2\sigma }_{CSE}\right)$ limits for the CSE database, promising extension of P and T waves detection for further VCG processing and automatic VCG classification.

## Data Availability

Publicly available datasets were analyzed in this study. This data can be found in the PTB Diagnostic ECG Database: https://physionet.org/content/ptbdb/1.0.0/. Requests to access the CSE database should be directed to prubel.lyon@gmail.com.
